# Damaged guidewire by the introducer needle tip while inserting central venous catheter in subclavian vein by supraclavicular approach

**DOI:** 10.4103/1658-354X.71576

**Published:** 2010

**Authors:** Rakesh Garg, Vijay Kumar Ramaiah, R.S. Chouhan

**Affiliations:** Department of Neuroanaesthesiology, Neurosciences Centre, All India Institute of Medical Sciences, New Delhi, India

Sir,

Central venous cannulation is now commonplace, but percutaneous catheterization of the subclavian vein was only introduced in 1952.[1] Seldinger technique, originally used to cannulate the vessels for radiographic procedures, is frequently used for central venous catheterization.[2] Various complications associated with guidewire use like knotting of the guidewire, partial or complete guidewire embolism, vascular injury while negotiating the ‘J’ tipped guidewire has been described earlier.[2–5]

Sharp beveled tip of introducer needle has been suggested to be the cause of ‘J’ tip being stuck while withdrawing the guidewire through the needle.[4] But damage to guidewire, proximal to the ‘J’ tip, due to sharp beveled end of introducer needle has not been reported.

A 22-year-old male weighing 48 kg was scheduled for midline sub-occipital craniotomy for posterior fossa space occupying lesion in sitting position. A week earlier, the patient has undergone vetricuo-peritoneal shunt insertion under general anesthesia uneventfully.

In the operating room, after attaching routine monitors, anesthesia was induced in a standardized manner and trachea was intubated. Ultrasound guided subclavian vein catheterization by supraclavicular approach using double lumen central venous catheter (CVC) (Multicath 2, Laboratoires Pharmaceutiques VYGON, B.P. 7-95440, ECOUEN – France) was planned. After positioning the patient, subclavian vein was identified in the supraclavicular region using the ultrasound (Terason t3000 CV ultrasound system, Teratech, Burlington, MA, USA) and the vein was punctured with the introducer needle at a depth of about 10 mm in two attempts. While introducing the guidewire through introducer needle, a slight resistance was felt but the guidewire could be negotiated to a required length. Slight resistance was again felt while withdrawing the introducer needle. The dilator was inserted over the guidewire again with slight resistance. After removing the dilator, a double lumen CVC was inserted over the guidewire but the catheter could not be negotiated after an insertion of about length 4 cm. The guidewire got stuck and could not be moved to and fro. The CVC along with guidewire was withdrawn simultaneously. The guidewire was observed to be damaged and its spirals got unwound from the central wire of the guidewire [Figure 1].

**Figure 1:**
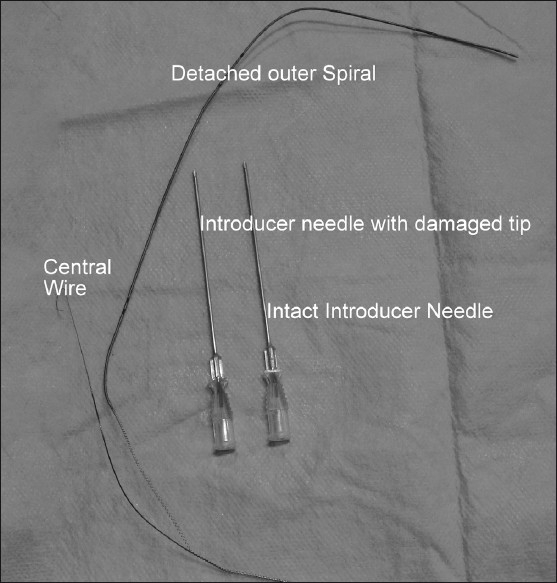
Damaged guidewire along with intact and damaged tipped introducer needle

Thereafter, CVC insertion through right internal jugular vein with a fresh set of guidewire and the surgical procedure were completely uneventful.

The impaction of ‘J’ tip of guidewire and its retrieval has been described,[4,6] but retrieval of impacted unwound spirals of guidewire has not been described earlier. We were fortunate in our case that the sharp J tip of the central wire did not damage the vein, and also it did not break completely and embolize distally.

The introducer needle has a sharp beveled end. During the supraclavicular approach for subclavian vein catheterization, introducer needle is inserted at an angle of about 30-40°. In our case, probably the guidewire could have been damaged while negotiating guidewire at the beveled end of the introducer needle. The chances are even more if the beveled end faces posteriorly as the guidewire would be in direct contact with sharp tip of the beveled end of the introducer needle. Any movement at this juncture could damage the spiral of the guidewire. The damage to guidewire was further aggravated by dilator in our case. The catheter got stuck with the unwound guidewire and so we were unable to negotiate the catheter to and fro.

Also, if the needle tip gets damaged as a consequence to its hitting the bone, then the chances of damage to the guidewire further increases. Damage of needle tip should be suspected incase it strikes any bony structure and further resistance is felt during guidewire/introducer needle/CVC insertion.

Safe use of guidewire for central venous access requires care in handling and understanding of their physical characteristics including its structure.[3] Monaca *et al*. have described the structure of a commonly used guidewire.[5] The guidewire consists of an inner single filament wire core and a surrounding coiled wire cover. The latter is designed as a helix of stainless steel to form a tunnel for the inner wire and provides elastic proper ties. Apart from the two ends of the guidewire where the outer spiral is welded to the inner wire, there is no further point of attachment between the core and the outer wire. Hence, any damage to the guidewire on its stem may lead to unwinding of the whole outer spiral as occurred in our case. Vascular injury while pulling back the guidewire by the sharp edge of unwound guidewire could occur.

We conclude that the bevel of the introducer needle should face anteriorly while inserting guidewire. Incase any undue resistance is felt, then the introducer needle and the guidewire should be withdrawn simultaneously without any excessive pull and inspected for any damage. Undue manipulation or application of force should not be applied while inserting dilator or central venous catheter.

## References

[1] McGoon MD, Benedetto PW, Greene BM (1979). Complications of percutaneous central venous catheterization: A report of two cases and review of the literature. Johns Hopkins Med J.

[2] Schummer W, Schummer C, Gaser E, Bartunek R (2002). Loss of the guidewire: mishap or blunder. Br J Anaesth.

[3] Khan KZ, Graham D, Ermenyi A, Pillay WR (2007). Case Report: Managing a knotted Seldinger wire in the subclavian vein during central venous cannulation. Can J Anesth.

[4] Arya VK, Kumar A (2004). Technique of retrieval of J-tip guidewire without withdrawing introducer needle during central venous cannulation by Seldinger technique. Anesth Analg.

[5] Monaca E, Trojan S, Lynch J, Dochn M, Wappler F (2005). Broken guidewire – a fault of design. Can J Anesth.

[6] Unnikrishnan KP, Sinha PK, Nalgirkar RS (2005). An alternative and simple technique of guidewire retrieval in a failed Seldinger technique. Anesth Analg.

